# Insufficiently complex unique-molecular identifiers (UMIs) distort small RNA sequencing

**DOI:** 10.1038/s41598-020-71323-0

**Published:** 2020-09-03

**Authors:** Klay Saunders, Andrew G. Bert, B. Kate Dredge, John Toubia, Philip A. Gregory, Katherine A. Pillman, Gregory J. Goodall, Cameron P. Bracken

**Affiliations:** 1 Centre for Cancer Biology, University of South Australia and SA Pathology, Adelaide, SA Australia; 2 Discipline of Medicine, The University of Adelaide, Adelaide, SA Australia

**Keywords:** Biological techniques, Computational biology and bioinformatics

## Abstract

The attachment of unique molecular identifiers (UMIs) to RNA molecules prior to PCR amplification and sequencing, makes it possible to amplify libraries to a level that is sufficient to identify rare molecules, whilst simultaneously eliminating PCR bias through the identification of duplicated reads. Accurate de-duplication is dependent upon a sufficiently complex pool of UMIs to allow unique labelling. In applications dealing with complex libraries, such as total RNA-seq, only a limited variety of UMIs are required as the variation in molecules to be sequenced is enormous. However, when sequencing a less complex library, such as small RNAs for which there is a more limited range of possible sequences, we find increased variation in UMIs are required, even beyond that provided in a commercial kit specifically designed for the preparation of small RNA libraries for sequencing. We show that a pool of UMIs randomly varying across eight nucleotides is not of sufficient depth to uniquely tag the microRNAs to be sequenced. This results in over de-duplication of reads and the marked under-estimation of expression of the more abundant microRNAs. Whilst still arguing for the utility of UMIs, this work demonstrates the importance of their considered design to avoid errors in the estimation of gene expression in libraries derived from select regions of the transcriptome or small genomes.

## Introduction

The counting of reads within high-throughput sequencing enables the relative abundances of transcripts to be compared between biological conditions. In order to do this, a PCR amplification step is typically required, both to amplify cDNA to a level sufficient for sequencing, and to enrich the molecules to which required adaptors have been successfully added. Biases in the PCR amplification step however lead to specific sequences being over- or under-represented in the final library^1^. This distorts the perceived depth of reads, which in turn introduces errors into downstream calculations such as transcriptome abundance or allele frequency in genomic data^2^.

One method to eliminate PCR bias is to remove all but one read of identical sequence, however the assumption that reads of the same sequence are a result of PCR bias is flawed, as it is dependent upon such factors as the depth of sequencing and the size of the genome/transcriptome being assessed. The collapsing of identical reads into singly-represented sequences is particularly problematic when assessing the transcriptional output of distinct subsets of the genome such as microRNAs (miRNAs), where their short lengths and tightly controlled biogenesis constrain the variety of sequences between independent molecules^3^.

To identify unique molecules in complex mixtures, the addition of random barcodes prior to PCR were proposed to make each individual molecule unique^4^. These barcodes, now referred to as Unique Molecular Identifiers (UMIs), enable PCR amplification to be performed with no loss of information, such that as long as library complexity is maintained, multiply represented reads of identical sequence can be de-duplicated to reveal the initial abundance of molecules^5,6^.

It has been noted that the complexity of potential UMIs must outweigh the number of independent identical molecules^3–5,7^, and investigation into the length of the barcode required to generate such diversity has indicated as few as 5^7^ or between 6–8 nucleotides^8,9^ is sufficient for genome and transcriptome sequencing applications.

With this in mind, we sequenced small RNAs (smRNAs) using a smRNA-specific sequencing kit (NEXTflex small RNA-Seq kit v3; Bioo Scientific) that utilise a UMI length of eight nucleotides, divided into 2 × 4 nucleotide sequences at the 5′ and 3′ end of the smRNA to be sequenced. Surprisingly however, we found that for several dozen of the most highly expressed miRNAs (that represent the vast majority of total miRNA expression) a shortage of available UMIs leads to an overly vigorous removal of duplicated reads and the resultant under-estimation of expression; up to more than 20 fold for the most highly expressed miRNAs. We present this data to inform researchers seeking to employ UMIs in the profiling of libraries of lower complexity, such as smRNA sequencing. We also discuss bioinformatic considerations when analysing UMI-containing libraries, including the effects of permitting single nucleotide mismatches within UMIs as is typically performed to accommodate PCR or sequencing error.

## Results

**Figure 1:**
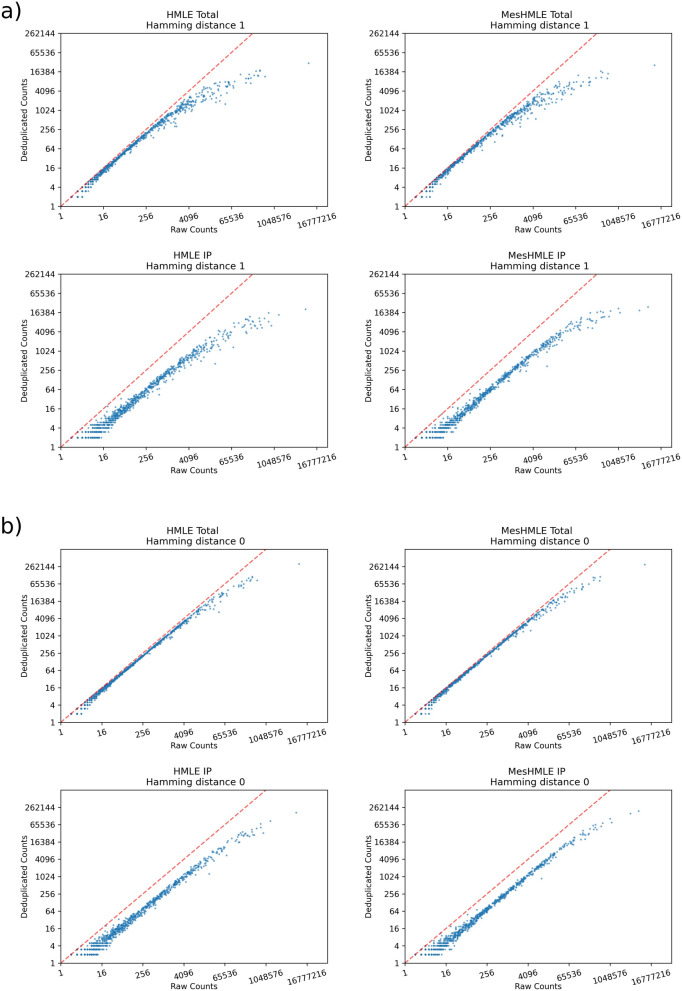
Highly expressed microRNAs are subject to over de-duplication. 4 separate smRNA libraries (HMLE and MesHMLE cells; total RNA and RNA co-immunoprecipitated with AGO) are shown, with each miRNA represented as dots plotted on axes of total RNA reads (x axis) and de-duplicated read counts (y axis). (**a**) Draws from a more limited pool of UMIs on account of de-duplicating otherwise identical reads in which there is a single nucleotide mismatch between UMIs (Hamming distance = 1), to account for PCR or sequencing error. In (**b**) no UMI sequence divergence is accommodated (Hamming distance = 0). For clarity, 4 libraries are represented here. Data from additional biological replicates are included in Supplementary Fig. 1.

**Figure 2:**
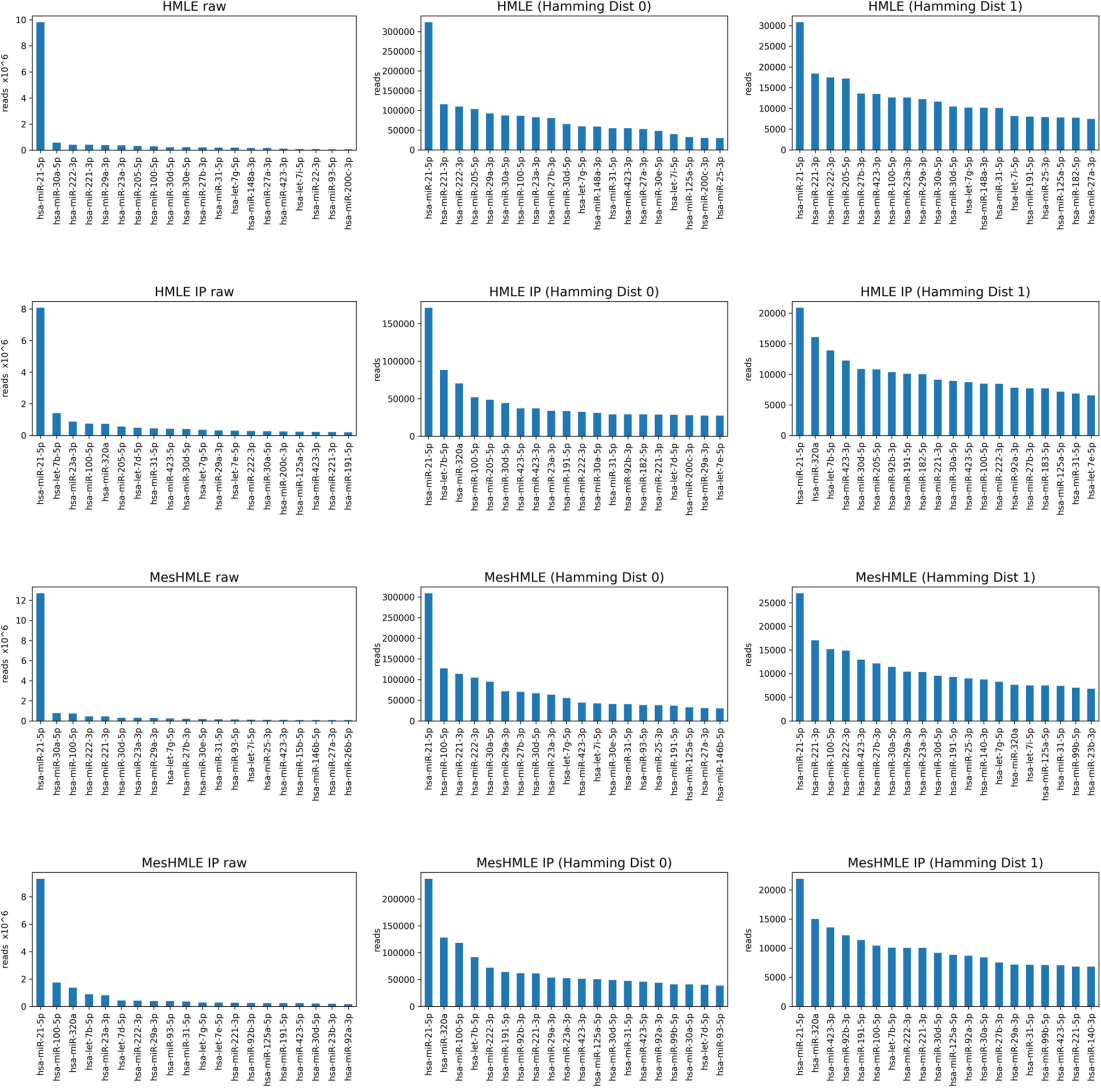
Over de-duplication due to limiting UMIs drastically decreases the apparent expression of more abundant miRNAs. MiRNA expression (counts per million) from raw reads or after de-deduplication (Hamming distance = 0 or 1) is shown for the top 20 miRNAs from each of the libraries analysed.

SmRNA sequencing was performed on libraries generated from epithelial HMLE cells and from mesenchymal derivatives of these cells (MesHMLEs) created via treatment with TGFβ (a promoter of Epithelial-Mesenchymal Transition, EMT, which the HMLE/MesHMLE system models)^10^. Both total smRNAs and smRNAs co-immunoprecipitated with Argonaute (AGO) were sequenced, with duplicates of identical sequence (including the 8 nucleotide UMI) discarded to eliminate PCR amplification bias. As sequencing and/or PCR errors are known to generate artefactual UMIs^9,11,12^, we initially analysed data accounting for a Hamming distance of 1, whereby otherwise duplicated reads that differ by one nucleotide within the UMI were subject to de-duplication. This method of assessment decreases the pool of available UMIs, which we found to be increasingly limiting with higher levels of miRNA expression (Fig. 1a, Supplementary Fig. 1a). In order to increase the pool of UMIs, we re-analysed the data not accounting for any UMI errors (Hamming distance of 0, Fig. 1b, Supplementary Fig. 1b). Although this decreased the observed over de-duplication (increasing the theoretical pool of potential UMIs from 16,384 to 65,536), this was not sufficient to eliminate the problem, resulting in the continued under-estimation of expression of several dozen of the most abundant miRNAs. This is a major concern given the top 10 miRNAs represent over 80% of all miRNA expression. In the most severe cases, over de-duplication, even when applying a Hamming distance of 0, under-estimated miRNAs by more than 20 fold (Fig. 2).

**Figure 3:**
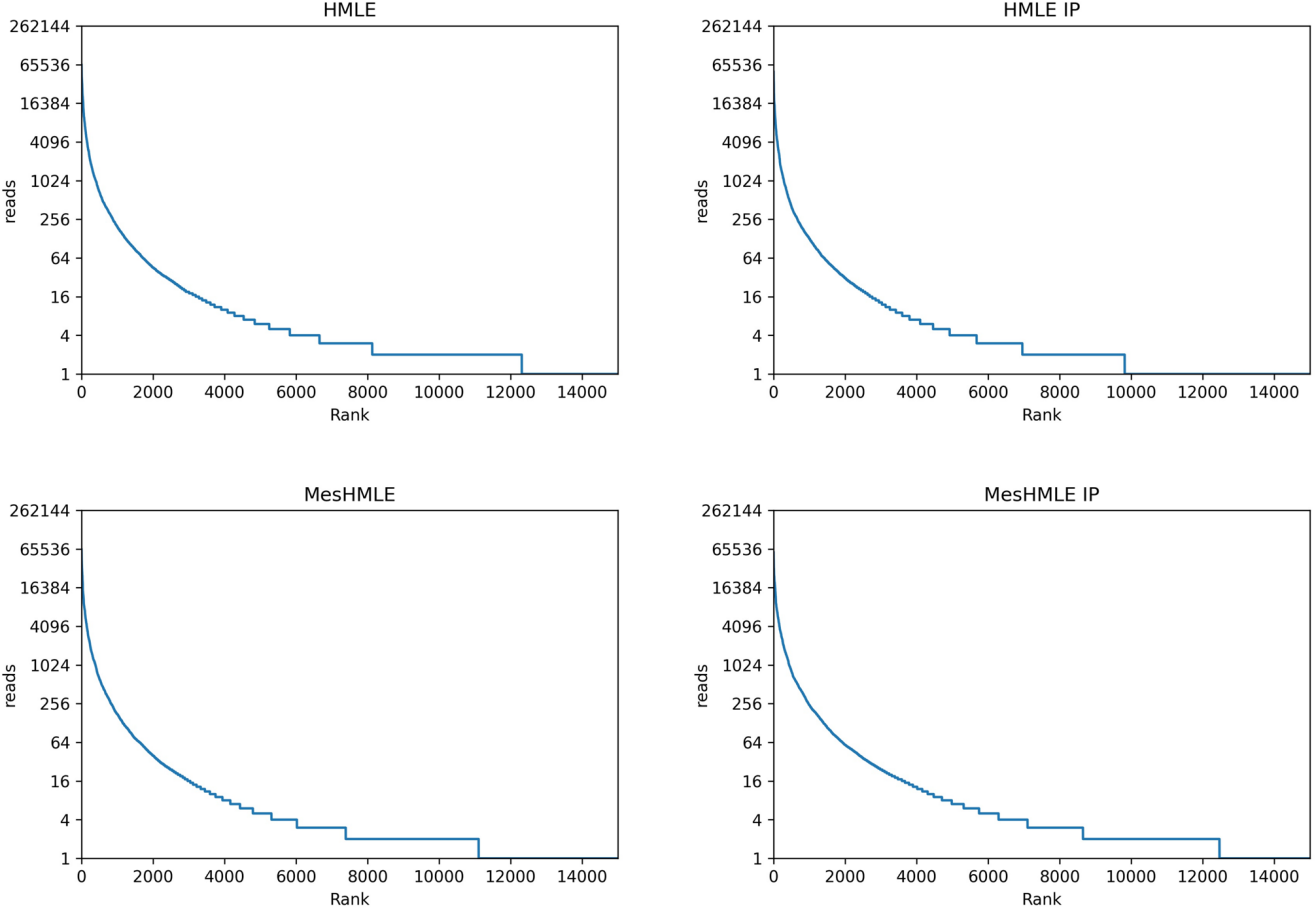
“Over-sequencing” is not responsible for limiting UMIs. Raw read counts of every isomiR detected were ordered and plotted to reveal the frequency of which individual molecules were sequenced.

Although the above data indicate that the number of UMIs available in a commercial smRNA sequencing kit are insufficient to adequately account for all miRNAs in the library, the extent to which this is a problem is dependent upon the depth of sequencing. If one were to over-sequence for example, an under-availability of UMIs would become exacerbated. To examine whether over-sequencing has contributed, we examined the number of times identical sequences were duplicated. In each of our libraries, thousands of molecules have only been sequenced once and in the RNA-Seq libraries, at least half of the UMIs were represented five or less times (Fig. 3). This indicates we have not over-sequenced our libraries and therefore, the problem of miRNA under-estimation after de-duplication is a function of insufficient complexity in the pool of UMIs, rather than the over-amplification of the libraries being examined.

**Figure 4:**
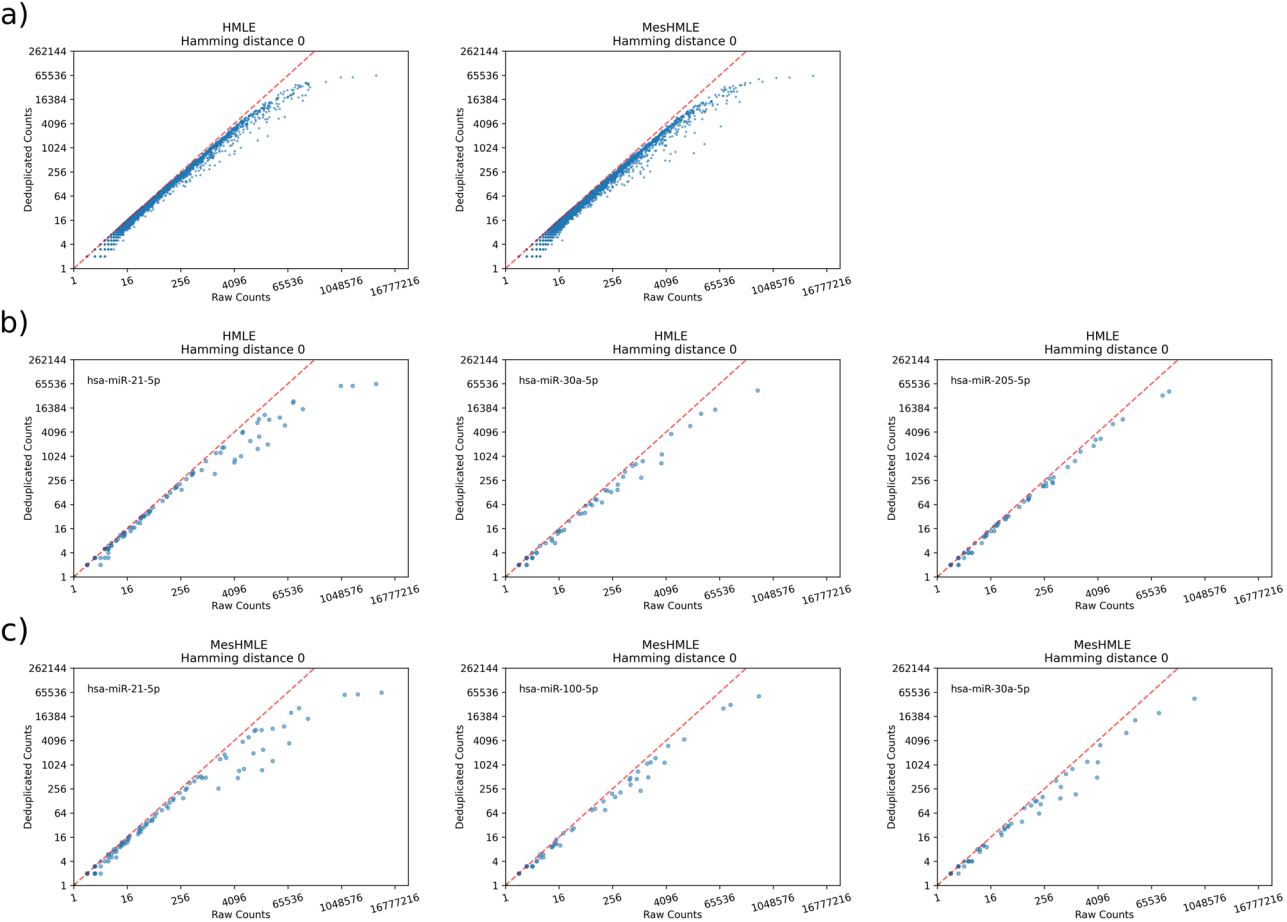
UMIs correct isomiR-specific PCR bias. (**a**) All isomiRs from HMLE and MesHMLE cells and all individual isomiRs of the 3 most expressed miRNAs in (**b**) HMLE and (**c**) MesHMLE cells are represented as dots plotted on axes of total RNA reads (x axis) and de-duplicated read counts (y axis, Hamming distance = 0).

**Figure 5:**
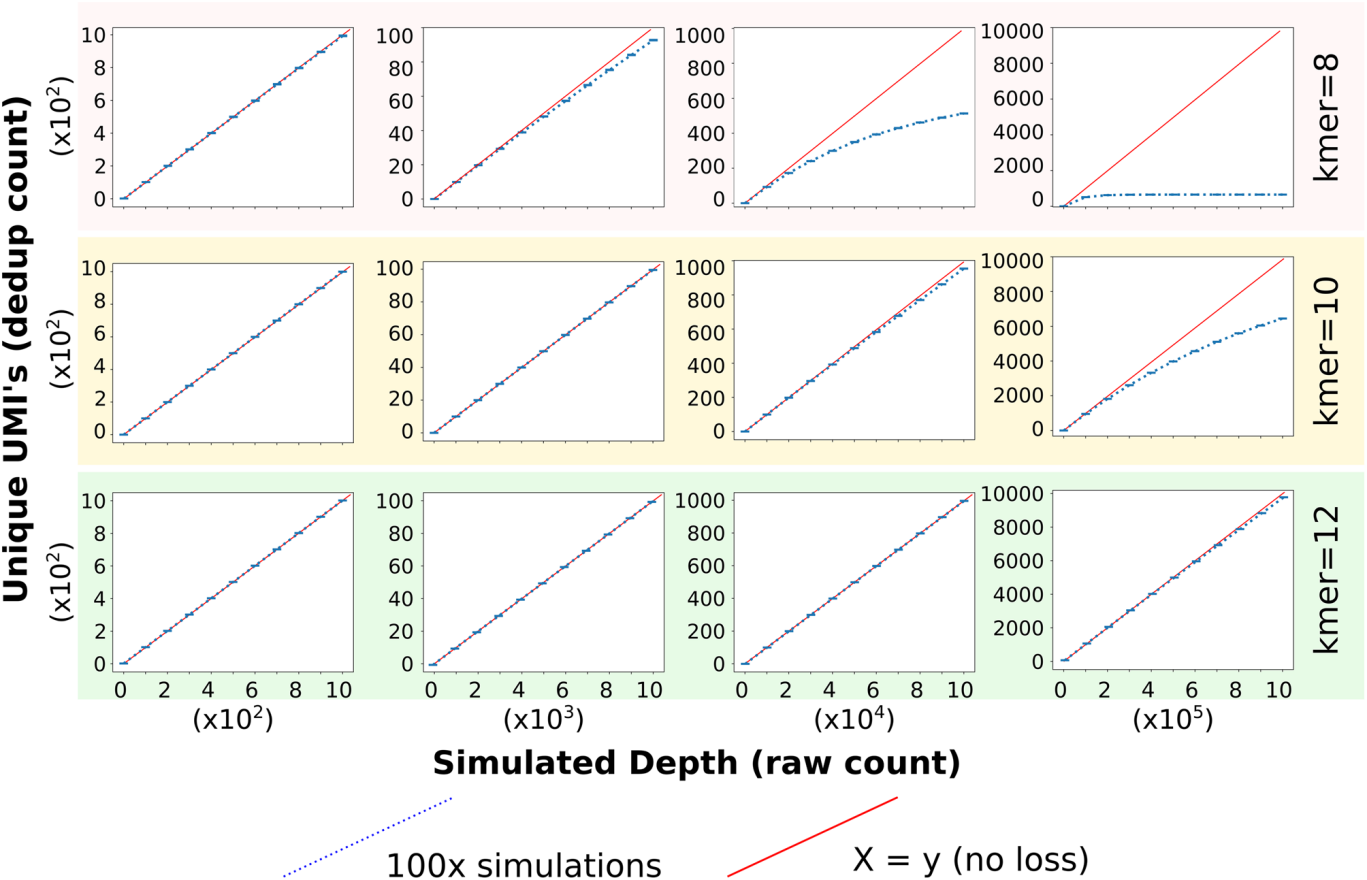
Simulation of de-duplication performance with differing UMI length and sequencing depth.

The quantitation of miRNAs from high-throughput sequencing is typically simplified through the condensing of similar reads from a common locus into a single read number that represents the expression of that specific miRNA. Whilst this simplification is often sufficient, it does eliminate the complexities of endogenous miRNA expression whereby individual miRNAs are often present as a series of naturally occurring sequence variants known as “isomiRs”^13^. Although typical processing of smRNA read data eliminates this layer of diversity, an increasing number of reports detail important functional differences between isomiRs^14–20^. When assessing miRNAs at the isomiR level, we note a consistent trend whereby over de-duplication remains problematic for all isomiRs expressed above a certain threshold (~ 50,000 total reads, Fig. 4a). Below this level of expression, PCR bias is uncommon, though there are consistent examples of specific isomiRs being over-amplified across samples (for example, miR-21-5p and miR-30a-5p, Fig. 4b,c), even where the expression of these molecules falls below the level at which UMIs become limiting. Taken together, this work argues for the employment of UMIs to eliminate PCR bias for the analysis of smRNA seq, especially at the isomiR level, though we also clearly demonstrate that a UMI sequence in excess of eight nucleotides will be required to resolve the relative expression levels of the more abundant miRNAs. Simulations of the performance of UMIs of different length in the context of variable sequencing depth are provided in Fig. 5. Based on this, a UMI length of 12 nt should provide sufficient coverage to avoid excessive de-duplication. We show this using data from small RNA libraries generated using a more recently released sequencing kit (QIAseq miRNA library) that employs a 12 nt UMI (Supplementary Fig. 2).

## Discussion

It has been reported that there is high variability in the read counts of smRNA libraries prepared using different methods/kits^21^. Biases, primarily in RNA ligation^22–24^ but also in PCR amplification^3^, represent sources for such variability. In an effort to reduce ligation bias, some methods include polyethylene glycol to improve overall ligation efficiency, and/or utilise random nucleotide sequences immediately adjacent to the miRNA ligation site to avoid sequence-specific differences. To account for differential PCR amplification, the incorporation of UMIs has proven a useful tool because individual cDNAs are uniquely tagged and can be identified after sequencing. This allows the researcher to amplify libraries to a level sufficient to detect low abundance transcripts, whilst retaining the original expression information because identical sequences that are a result of the library preparation can be collapsed to count as a single read. Thus, gene expression changes will reflect true biological differences. The use of UMIs for smRNA-Seq has been reported to decrease variance between technical replicates and to increase both accuracy and sensitivity of miRNA detection^3,21^.

For most genomic or transcriptomic applications for which there is massive complexity, only short UMI sequences are required. For example, when using a five nucleotide UMI, not one single saturated position was identified in the transcriptomic sequencing of mouse embryonic stem cells^7^. For a library of more limited complexity however, such as for miRNA-sequencing in which there is a more restricted range of possible sequences, we find that a more diverse array of UMIs are required. We find that even when using a library-preparation kit specifically designed for smRNA-sequencing (NEXTflex small RNA-seq kit v3) that incorporates eight nucleotide identifiers, the amount of UMIs became limiting for more highly expressed miRNAs. This is problematic, as the employment of UMIs to reduce PCR bias will inadvertently cause the under-estimation of expression of the more highly abundant miRNAs, thus creating the false impression that there is relatively similar levels of expression between many miRNAs whereas in reality, the few most abundant miRNAs represent the majority of total miRNA expression (Fig. 2).

In agreement with other recent studies^3,25,26^, we found a strong correlation between UMI counts and raw read counts, suggesting relatively little PCR bias exists within our samples. This suggests one might dispense with UMIs altogether in smRNA sequencing, though for low concentration samples that require more extensive amplification, we have found UMIs are helpful to maintain information of relative miRNA expression (data not shown). Further arguing for the application of UMIs, we also note isomiR-specific PCR biases occur, and may be relatively prominent in isolated examples (such as miR-21-5p or miR-30-5p). Given a growing body of evidence suggesting the functional importance of isomiRs^14–20^, an argument can be made that miRNA expression should be analysed at the isomiR level, rather than aggregating all variants into one miRNA read count. The incorporation of UMIs will increase the accuracy of this, though as we show for libraries of limited complexity sequenced at sufficient depth, increased UMI diversity beyond eight nucleotides is required for an unbiased estimation of expression.

## Methods

### Tissue culture

HMLE cells^10^ were cultured in HuMEC Ready Media (ThermoFisher) and induced to undergo EMT by transferring to DMEM:F12 media (1:1) supplemented with 10 lg*/*ml insulin, 20 ng*/*ml EGF, 0.5 lg*/*ml hydrocortisone and 5% fetal calf serum (FCS) and treating with 2.5 ng*/*ml of TGF-β1 (R&D) for at least 14 days. MesHMLE cells, which are derived from HMLE from prolonged treatment with TGF-β1, were maintained in EMT-inducing media without additional TGF-β1.

### Small RNA-seq methods

HMLE and MesHMLE cells were grown in 100 mm plates to ~ 90% confluency, rinsed once with ice-cold PBS, and total RNA isolated using TRIzol reagent (Invitrogen) from two plates per cell line. RNA was treated with TurboDNase (Invitrogen) followed by extraction with acid phenol (ThermoFisher, AM9712)/chloroform and precipitation with 1:1 ethanol:isopropanol. Purified RNA was quantitated by nanodrop and Bioanalyser before 1 µg RNA was used to generate small RNA libraries using a Nextflex small RNASeq kit (Bioo) with 14 cycles of amplification. PCR products were separated on an 8% acrylamide (19:1) TBE denaturing gel, stained with SYBR Gold nucleic acid gel stain (ThermoFisher) and imaged on a ChemiDoc MP (BioRad). Products corresponding to an insert size of 21–27 nt were excised from the gel and extracted by the “crush and soak” method as previously described^27^. Library quality and quantity was assessed by Bioanalyzer (Agilent), Qubit (ThermoFisher) and qPCR using the NEBNext Library Quant kit for Illumina (NEB). Equivalent amounts of each library were pooled and sequenced on an Illumina NextSeq 500 (1 × 75 bp).

### Bioinformatic processing

Sequencing adapters were removed from small RNAseq reads using Cutadapt (v2.8)^28^. Cutadapt command line parameters: adapter = TGGAATTCTCGGGTGCCAAGG; error rate = 0; overlap = 5; minimum length = 18. In order to remove the two 4 nt bioo UMIs, UMI-tools (v1.0.0) was used twice to cut the 5′ UMI and 3′ UMI from reads^9^. Reads shorter than 18 nt after adapters and UMIs had been trimmed were discarded. The remaining reads were mapped to the Human Genome (*Homo sapiens* version hg19, from UCSC) using BWA backtrack algorithm (v0.7.15)^29^. Default parameters for both the maximum edit distance (−n 0.04) and maximum difference in the seed (−k 2) were used. Alignments are then de-duplicated using UMI-tools with the parameter edit-distance-threshold equal to 0 or 1 depending on the desired output. MicroRNA annotation (*Homo sapiens* version hg19, from UCSC) and HTSeq-count (v 0.9.1) was used for microRNA level expression of samples^30^. The sum of all unique reads was counted for each microRNA locus and the mean multimapping read count of all loci were summed to calculate the total microRNA expression. Isomir level expression was calculated using custom scrips (https://bitbucket.org/sacgf/saunders_umi_2020/src/master/). For microRNAs with multiple loci, a representative locus is selected and isomirs counted based on the start and end positions relative to the microRNA annotation. Read counts for all libraries are presented in Supplementary Table 1.

### Simulating the effect of UMI size on deduplication (sampling from an infinite population)

In order to determine empirically the depth at which one expects to see a single UMI (of a given length) attached to two or more unique starting molecules in a sequencing library, we simulated the problem. To do so, we first created a complete set of UMIs composed only of the letters *A*, *C*, *G* and *T*, and of length *L *(where *L* = 4 data not shown, 8, 10 or 12). The complexity of the full set of available UMIs (*C*) is dependent on *L* and increases exponentially as *L* increases. For instance, when *L* = 4, *C* = 4^4^ = 256; when *L* = 8, *C* = 4^8^ = 65,536 and when *L* = 12, *C* = 4^12^ = 16,777,216. We then simulated the ligation of UMIs to unique molecules in a starting sample by randomly selecting from the entire population *n* times and recording each selection. In selecting UMIs, we assume that all unique sequences of length *L *in the population of UMI’s, called kmers (*k*), are infinitely abundant and equally likely to be chosen. These assumptions are valid for sequenced samples when the number of UMI’s available far outnumber that of sequenced molecules, which is usually the case. After predefined numbers of selection and multiple rounds of simulation (100), we investigate the unique number of UMIs extracted. Clearly, when *C* is large and *n* is small, the probability of selecting the same UMI more than once is close to 0. However, as the number of selections increases and becomes equal or overtakes the number of unique elements in the population, the probability of selecting the same UMI increases likewise, approaching 1. By counting the unique number of UMIs after *n* selections in the simulated data and plotting the results, the distance between the simulated line (blue) and the expected line when no *k* is selected twice (red: x = y) represents the number of unique UMI’s extracted more than once. The simulation shows that when *L* = 8, after only 10,000 selections, a visible separation can be seen between the simulated line and the expected line when no *k* is selected twice. This implies that at this depth, given this length of UMI, the likelihood of selecting the same UMI more than once becomes apparent.

The probability of selecting a particular UMI two or more times can easily be derived analytically, namely

$$\mathrm{P }= 1{-}\mathrm{Pr}\left(\mathrm{UMI not being sampled}\right) {-}\mathrm{Pr}\left(\mathrm{UMI being sampled exactly once}\right)$$

$$=1-{\left(\frac{C-1}{C}\right)}^{n}-n*\frac{1}{C}*{\left(\frac{C-1}{C}\right)}^{n-1}.$$

For example, when *C* = 65,536 (*L* = 8) and *n* = 10,000 this becomes P = 0.125. Thus, the total number of UMI’s sampled more than once given these parameters is simply P times the number of UMIs available, i.e. P *C* = 689.5, which matches the simulation.

## Supplementary information

Supplementary Information.
