# Supplementary material for: Insufficiently complex unique-molecular identifiers (UMIs) distort small RNA sequencing
Source: Sci Rep. 2020 Sep 3;10:14593. doi: 10.1038/s41598-020-71323-0 (PMC7471316; doi:10.1038/s41598-020-71323-0)
Supplement: Supplementary file 1 — Supplementary Information. [file 41598_2020_71323_MOESM1_ESM.pdf]

SUPPLEMENTARY INFORMATION

Insufficiently complex Unique-Molecular Identifiers (UMIs) distort small RNA sequencing

Klay Saunders[1]; Andrew G Bert[1]; Kate B Dredge[1]; John Toubia[1]; Philip A Gregory[1,2]; Katherine A Pillman[1]; Greg J Goodall[1,2] and Cameron P Bracken[1.2]

[1]Centre for Cancer Biology, University of South Australia and SA Pathology, Adelaide, SA, Australia

[2]Discipline of Medicine, The University of Adelaide, Adelaide, SA, Australia

Supplementary Figure 1 – Highly expressed microRNAs are subject to over de-duplication

As with Figure 1, miRNAs (represented as dots) from smRNA libraries are plotted on axes of total RNA reads (x axis) and de-duplicated read counts (y axis). 6 samples in total (2 HMLE, 4 MesHMLE) were assessed. Each sample is paired (total RNA Seq on top and AGO IP below). Hamming distance = 1 and = 0 are represented in Supplementary Figures 1a and 1b respectively.

Supplementary Figure 2 – MicroRNA reads are not subject to over de-duplication when a longer (12nt) UMI is utilised

MicroRNAs were sequenced using a QIAseq miRNA library kit that employs a 12nt UMI length. Hamming distances = 1 and = 0 are shown on top and below respectively. Data taken from Wong et al (26).

Supplementary Table 1 – Read sequencing and mapping numbers for each library from Supplementary Figure 1

Supplementary Figure 1a

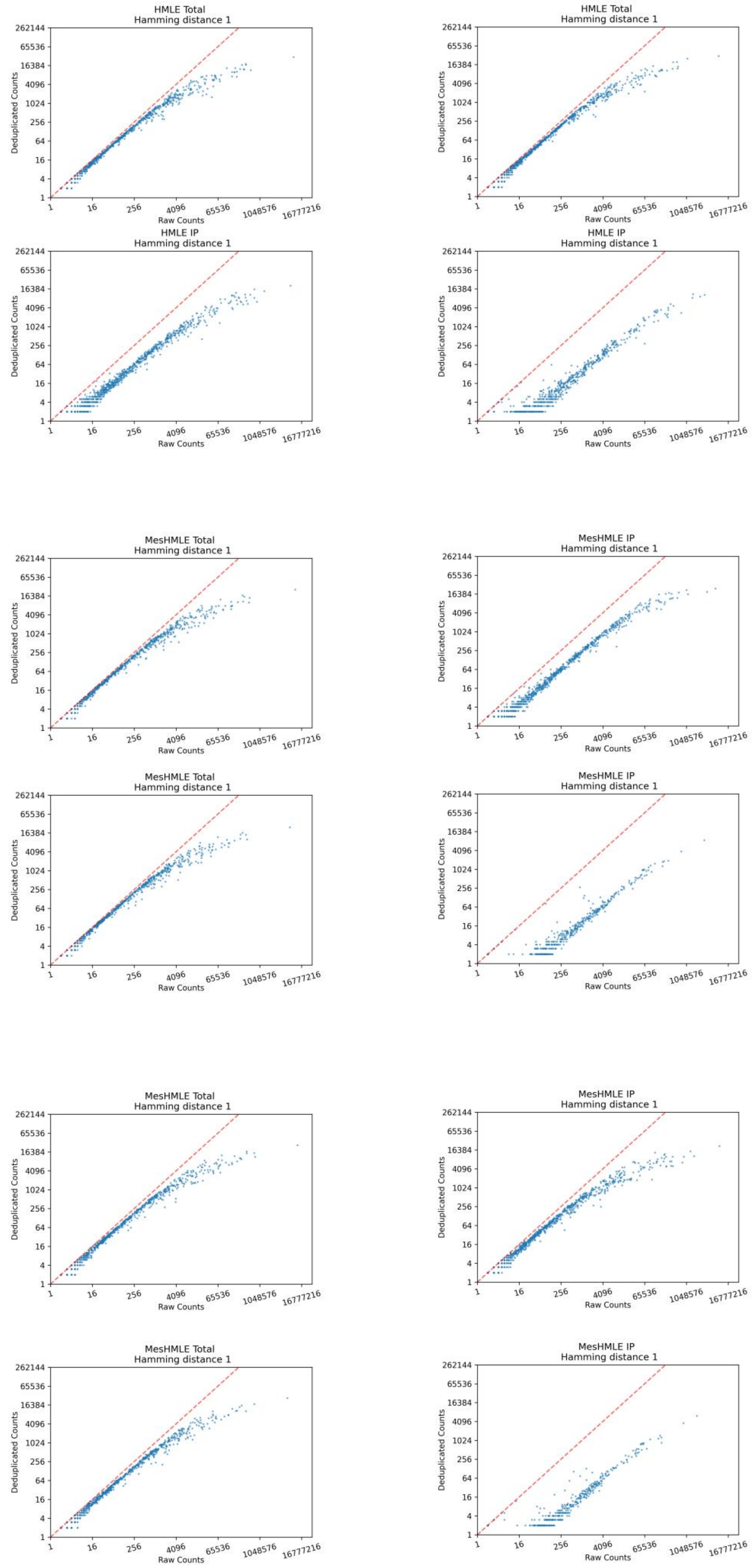

Supplementary Figure 1b

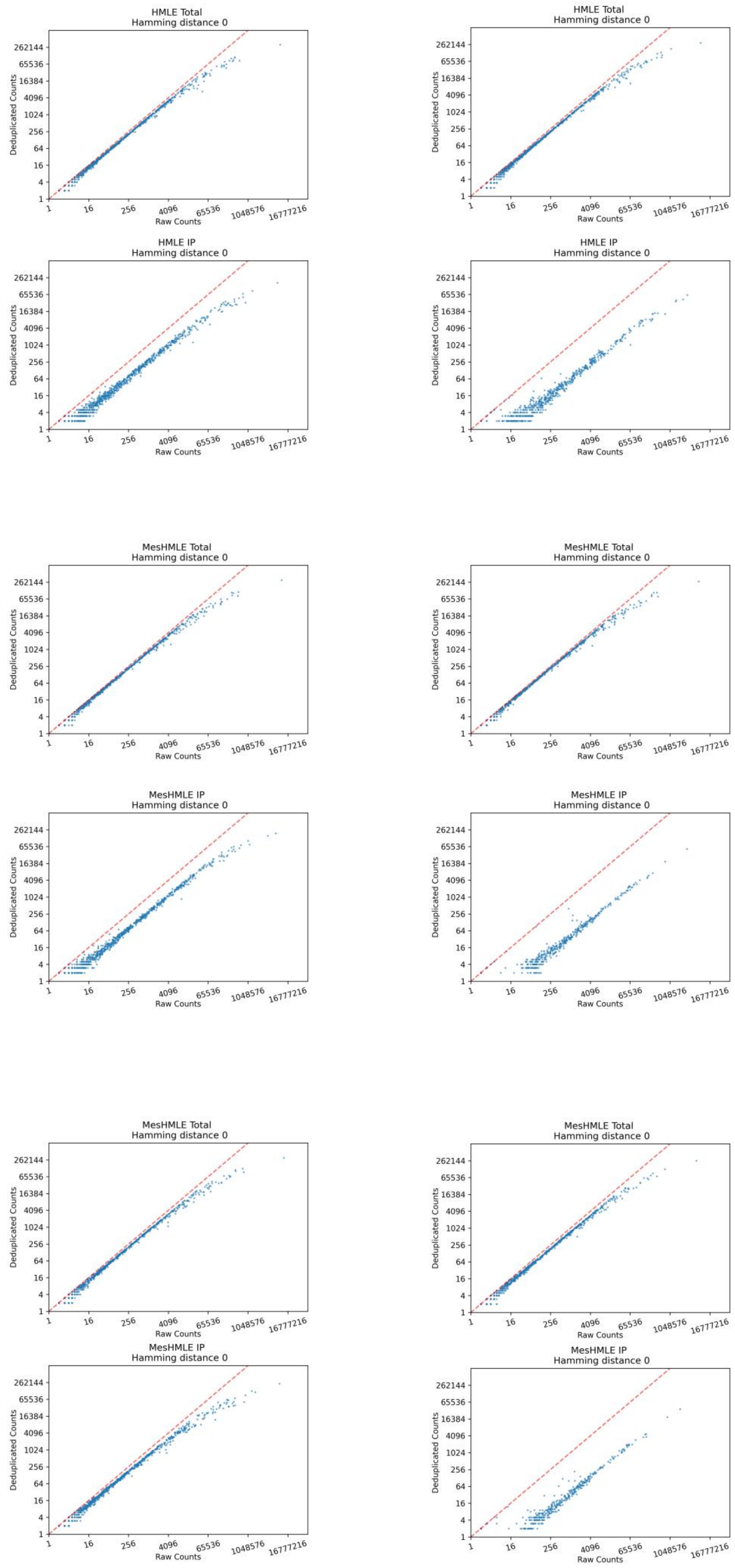

Supplementary Figure 2

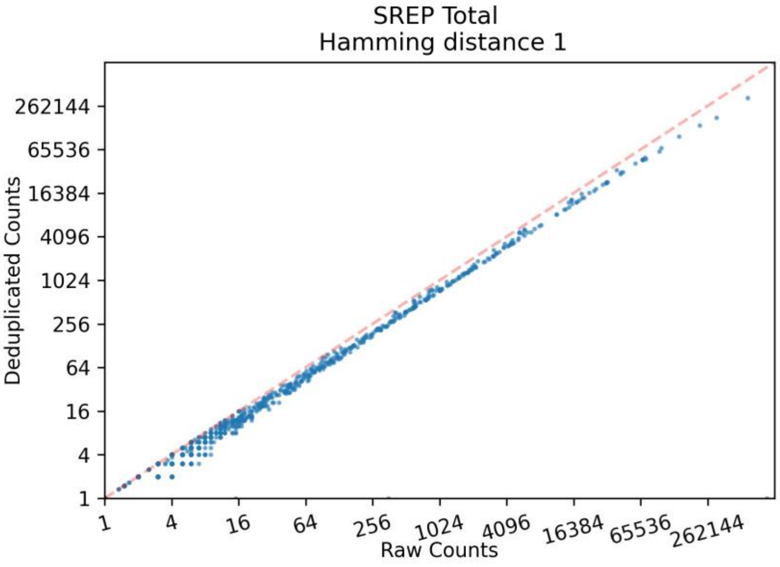

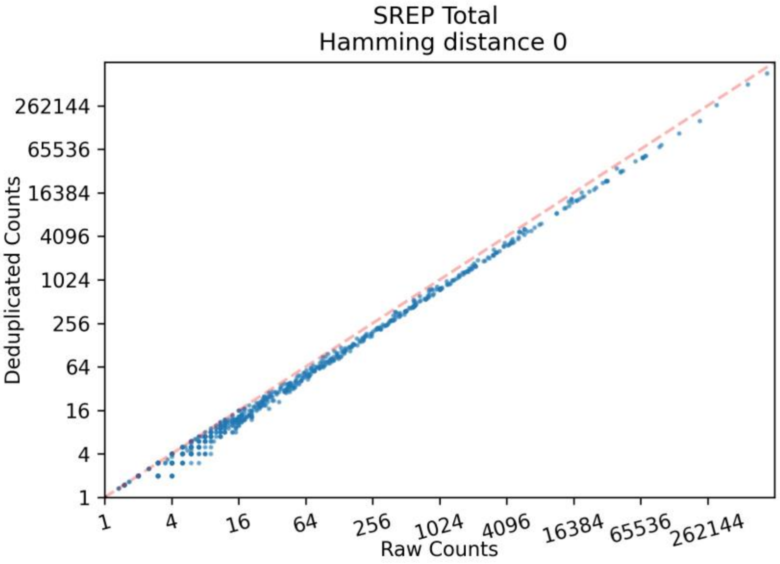

Supplementary Table 1

Sample	Total sequences	Mapped	% mapped	UMI dedup (ed=0) Deduped	# lost	% lost	UMI dedup (ed=1) Deduped	# lost	% lost
**H1**	23,307,989	22,911,560	98.30%	2,208,585	28,389,113	92.78%	610,065	29,987,633	98.01%
**H1_IP**	31,103,367	30,597,698	98.37%	5,935,489	16,976,072	74.09%	2,607,465	20,304,096	88.62%
**H5**	31,016,453	29,993,663	96.70%	1,227,002	24,573,240	95.24%	559,215	25,241,027	97.83%
**H5_IP**	26,694,707	25,800,242	96.65%	10,848,489	19,145,176	63.83%	5,653,782	24,339,883	81.15%
**M2**	21,775,030	21,293,910	97.79%	3,405,022	29,690,097	89.71%	1,194,899	31,900,220	96.39%
**M2_IP**	33,472,271	33,095,119	98.87%	4,404,438	16,889,472	79.32%	1,613,619	19,680,291	92.42%
**M3**	16,630,539	16,294,053	97.98%	640,552	11,675,484	94.80%	376,281	11,939,755	96.94%
**M3_IP**	13,086,696	12,316,036	94.11%	3,865,215	12,428,838	76.28%	1,369,609	14,924,444	91.59%
**M4**	25,550,111	25,173,554	98.53%	3,014,270	29,263,430	90.66%	631,894	31,645,806	98.04%
**M4_IP**	32,595,664	32,277,700	99.02%	4,354,658	20,818,896	82.70%	1,519,713	23,653,841	93.96%
**M5**	14,759,855	14,523,946	98.40%	368,943	8,033,036	95.61%	203,268	8,198,711	97.58%
**M5_IP**	8,701,266	8,401,979	96.56%	3,025,323	11,498,623	79.17%	1,100,216	13,423,730	92.42%
